# Fasting elicits gut microbiome signature changes that extend to type 1 diabetes patients

**DOI:** 10.3389/fendo.2025.1623800

**Published:** 2025-08-13

**Authors:** Franziska A. Graef, Bettina Berger, Lina S. Bahr, Rainer Stange, Andreas Michalsen, Friedemann Paul, Bruce A. Vallance, Kevan Jacobson

**Affiliations:** ^1^ Department of Pediatrics, BC Children’s Hospital, University of British Columbia, Vancouver, BC, Canada; ^2^ Chair of Medical Theory, Integrative and Anthroposophic Medicine, Faculty of Health, Witten/Herdecke University, Witten, Germany; ^3^ NeuroCure Clinical Research Center and Department of Neurology, Charité - Universitätsmedizin Berlin, corporate member of Freie Universität Berlin, Humboldt Universität Berlin, Berlin, Germany; ^4^ Institute of Social Medicine, Epidemiology & Health Economics, Charité - Universitätsmedizin Berlin, Berlin, Germany; ^5^ Department of Internal and Integrative Medicine, Immanuel Hospital Berlin, Charité – Universitätsmedizin Berlin, Berlin, Germany; ^6^ Experimental and Clinical Research Center, a cooperation between Charité - Universitätsmedizin Berlin and Max Delbruck Center for Molecular Medicine, Berlin, Germany

**Keywords:** type 1 diabetes, fasting, therapeutic fasting, gut microbiome, gut bacteria, nutrient availability, autoimmunity, multiple sclerosis

## Abstract

The gut microbiome has been linked to the pathogenesis of type 1 diabetes (T1D), identifying it as a promising therapeutic target. Nutritional interventions, which are an effective way to modulate the gut microbiome, thus show potential to be applied as complementary therapies for T1D. One particular dietary intervention, prolonged therapeutic fasting, has been shown to ameliorate symptoms of several autoimmune diseases, while also modifying the gut microbiota composition of healthy populations. It is unclear, however, how the gut microbiota of patients suffering from diseases of autoimmunity will respond to fasting. In this pilot study, we investigate the effects of prolonged fasting on the gut microbiome of T1D patients: Fasting substantially changed the composition and structure of the T1D gut microbiome so that it converged with that of non-diabetic controls immediately post fasting. Moreover, a comparison with a population of patients suffering from Multiple Sclerosis revealed substantial overlap in post-fasted microbiome changes and a remarkable consistency with published data of non-autoimmune populations, indicating that fasting leads to signature microbiome changes that are independent of host health status and disease type. A correlation analysis between fasting-mediated microbiota modifications and changes in clinical parameters revealed several significant associations between the *Oscillospiraceae* and *Lachnospiraceae* families and cholesterol and blood pressure changes in the T1D cohort, corroborating previous studies reporting on these associations in non-diabetic subjects. In conclusion, the observed fasting-mediated microbiome signature suggests that nutrient availability is a major disease-independent factor in shaping gut microbiome composition, likely driven by the need for metabolic diversification of microbial nutrient acquisition. The corresponding clinical associations highlight the need to investigate if these fasting-driven changes in the reported taxa are causally linked to the recorded clinical benefits of therapeutic fasting and what importance fasting as an additional therapeutic intervention might have to improve long term conditions in people with T1D.

## Introduction

Type 1 diabetes (T1D), a condition marked by the autoimmune destruction of the insulin-producing pancreatic β-islet cells, is estimated to affect 9 million people globally and involves life-long insulin replacement therapy, complex dietary planning and places patients at an elevated risk of developing co-morbidities, altogether signifying a high disease burden for this population (1, 2). The etiology of T1D is complex, several environmental and genetics risk factors are known: Studies have identified specific genetic loci conferring a heightened risk for disease development (3). However, in Western societies, the incidence and prevalence of T1D have been significantly increasing over the past 30 years pointing to environmental factors playing a key role in disease pathogenesis (4–6).

Indeed, the gut microbiota has emerged as an important environmental factor linked to autoimmune disease onset and progression (7). The gut microbiome of patients suffering from T1D has been shown to differ from that of healthy controls in both composition and functional capacity (8). Notable changes in intestinal microbiome composition have been reported to precede T1D onset (9). In addition, patients suffering from, or at risk of developing T1D, display increased gut permeability as compared to healthy controls (10). Moreover, many known risk factors for autoimmune diseases, such as mode of birth delivery, diet, infections, early-life antibiotic exposure, and psychological stress, also impact gut microbiota composition and function (11). Studies have shown that children delivered by caesarian section or who received antibiotics in their first two years of life, and as a result at least transiently carried a perturbed microbiota, are at higher risk for developing autoimmune diseases, including T1D (12–14).

These links between the microbiota and autoimmunity may reflect a loss of beneficial gut bacteria along with the metabolites they produce, such as short chain fatty acids (SCFA), retinoic acid, or amino acid metabolites including tryptophan. Microbial metabolites are critical for gut health as they modulate epithelial and immune cell function and maturation. For example, multiple bacterial compounds induce and fine-tune the balance between anti-inflammatory regulatory T (Treg) and pro-inflammatory Th17 cells or critically support the integrity of the gut barrier (15). Conversely, these early-life interventions can also favor the expansion of microbes with pathogenic qualities that may heighten the intestinal inflammatory tone of their host (16). Moreover, several gut bacteria have been identified that express epitopes resembling self-antigens (17). When these microbes activate the immune system, the resulting microbe-targeting T cells and/or antibodies can prove auto-reactive, targeting and damaging host cells. This “molecular mimicry” has been observed with the gut bacterium *Parabacteroides distasonis* which carries a sequence closely related to a key insulin epitope, with its presence accelerating T1D in a mouse model, while also being associated with T1D development in a pediatric population (18).

Current treatment options for T1D largely focus on continuous insulin substitution therapy and delaying β-cell loss by suppressing the aberrant immune response i.e., via immune modulators or biologicals (19). These therapies, especially the immunomodulators, are often expensive, require life-long administration, and can come with adverse side effects (20).

Since inflammation is metabolically demanding and immune cells respond to metabolic signals, another promising strategy to attenuate autoimmunity is to restrict metabolic fuel, through dietary interventions such as a therapeutic fast (21). Moreover, changes to the host diet are among the quickest and most impactful ways to modulate the gut microbiome (22). Consequently, nutritional interventions are uniquely suited as complementary, adjuvant immunotherapy (23).

Traditionally dietary interventions, especially fasting and other restrictive diets, have been more challenging to implement in people with T1D as the disease limits the patients’ metabolic flexibility. However, as technology and disease management options improve, adjuvant dietary modifications are becoming more feasible (24). For example, Ramadan fasting in T1D patients meeting pre-defined clinical criteria, appears to be safe (25). Furthermore, the absence of severe adverse events such as diabetic ketoacidosis in T1D patients undergoing a medically supervised seven-day fast, indicates the feasibility of a fasting intervention for this patient population (26).

Indeed, fasting has been shown to suppress both innate and adaptive immune pathways in mouse models and humans (27, 28). Promising studies in mice show that repeated cycles of a fasting mimicking diet (FMD) not only decrease the number of auto-reactive T cells in models of T1D but also promote the proliferation of Treg cells and the regeneration of pancreatic islet β cells (29). Notably, these fasting-mediated benefits are also seen in the context of other autoimmune diseases. For example, research examining the effect of fasting and FMD on experimental immune encephalitis (EAE) reported analogous results (30). EAE is used as a model for multiple sclerosis (MS), a neurodegenerative disease of the central nervous system, characterized by chronic inflammation and demyelination, affecting 2.8 million people worldwide (31). In the EAE model, fasting was shown to improve the T cell profile (decreased effector T cells and increased Treg cells) while spurring the regeneration of oligodendrocytes (30).

Still, the mechanisms by which fasting mediates its beneficial effects on autoimmune diseases are incompletely understood. While microbiome-independent effects on immune cells are undoubtedly involved (32, 33), several studies in mice have implicated the gut microbiome. For example, one study showed that β-hydroxybutyrate, the ketone body that is produced and used as the main fuel during fasting, depletes *Bifidobacteria* in humans and mice (34), with *Bifidobacteria* previously shown to induce intestinal Th17 cells, which play a role in T1D pathogenesis (35, 36). Indeed, the ketone-mediated decrease of *Bifidobacteria* was proven to be responsible for a reduction in intestinal Th17 cell proliferation (34). Furthermore, fecal microbiota transfer (FMT) from mice that were intermittently fasted conferred protection against EAE in recipient mice (37) and from DSS-induced colitis in transplanted mice (38). To date, several studies have examined the effects of prolonged fasting on the gut microbiome in humans (39). One study reported that a 10 day fast reduced the abundance of carbohydrate degrading bacteria like *Lachnospiraceae* and *Ruminococcaceae* while increasing the abundance of *Escherichia coli* and *Bilophila wadsworthia*, which are known to utilize host-derived nutrients (40). Similarly, a clinical trial in patients with hypertensive metabolic syndrome found that a 5 day fast significantly altered the metabolic capacity of the gut microbiota (41), enriching gene modules for propionate production, mucin degradation and other nutrient utilization pathways (41). Other fasting studies report similar results including an increase in the mucin-degrading species *Akkermansia muciniphilia* at both 3 days and 6 weeks after a fast (39, 42).

Converging evidence from these studies suggests that limiting dietary nutrients favors gut microbial community members with broader metabolic capabilities, enhancing their capacity to harvest host-derived sources of energy (43, 44). However, it is currently unclear how prolonged fasting affects the gut microbiome in patients with T1D, i.e. whether disease-specific changes arise or whether the impact is comparable to that of controls and to the literature at large. We explored this question by analyzing the metagenomic composition of the gut microbiome in study participants with and without T1D following a prolonged fast. We found that fasting led to pronounced yet transient changes in gut microbiome composition in T1D patients. Moreover, we report that specific microbiota members, mainly of the *Oscillospiraceae* and *Lachnospiraceae* families, show links with fasting-induced changes of cardiometabolic risk markers, especially cholesterol and blood pressure changes. We additionally analyzed the gut microbiome of a small cohort of patients (n=10) suffering from MS and following repeated prolonged fasts using this opportunity to search for potential common or disease-specific microbial patterns. We identified an overlap in the differentially abundant bacteria driving the post-fasting microbiome changes, within MS patients undergoing repeated fasts as well as between MS and T1D patients and controls, indicating the existence of a disease-independent microbiome fasting signature.

## Results

### Study and participant characteristics

**Figure 1:**
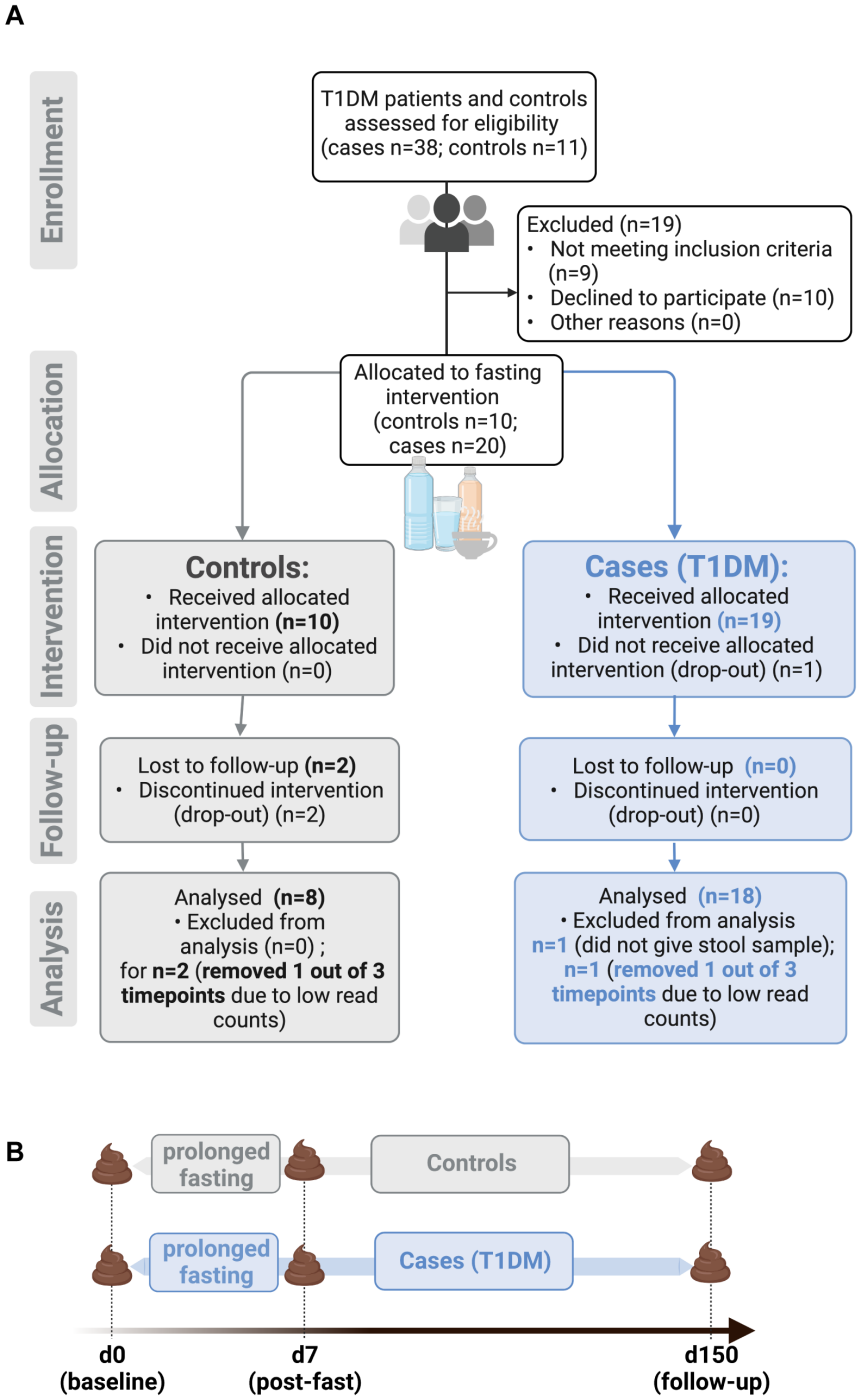
Overview of study design for FAMED1 study. **(A)** Prolonged fasting for patients with type 1 diabetes mellitus (FAMED1) is a non-randomized controlled pilot study that enrolled a total of 30 people. **(B)** Timeline of stool sample collection. Figure created with BioRender.com.

Stool samples were obtained from a controlled pilot study assessing safety and feasibility of prolonged fasting for patients with T1D (FAMED1) (**Figure 1A**). Stool specimens were collected at baseline (day 0), immediately after the seven-day fasting period (d7) and at follow-up (d150) (**Figure 1B**). Baseline characteristics of the study cohort (T1D n=19, 95% female; controls n=10, 70% female) aside from BMI (T1D 27.68 ± 7.04 kg/m^2^; controls 26.20 ± 4.74 kg/m^2^ - mean ± SD) and age (T1D 56.4 ± 6.2 years; controls 45.7 ± 13.8 years - mean ± SD) were previously published, along with inclusion and exclusion criteria (26). In brief, only patients with diagnosed T1D for ≥ 2 years were included (average disease duration 34.3 ± 3.54 years - mean ± SD), while patients with severe disease such as malignancies, kidney failure and infections were excluded (26, 45). The clinical outcomes for this study showed that fasting-related adverse effects were mild and temporary, with no events of diabetic ketoacidosis occurring in patients with T1D. Moreover, markers of metabolic health such as BMI and LDL-to-HDL ratio improved significantly throughout the fasting and follow-up period, demonstrating that fasting performed under medical supervision is safe and could prove beneficial for patients with T1D (26).

### Convergence of post-fasting gut microbiome composition of T1D patients and controls

**Figure 2:**
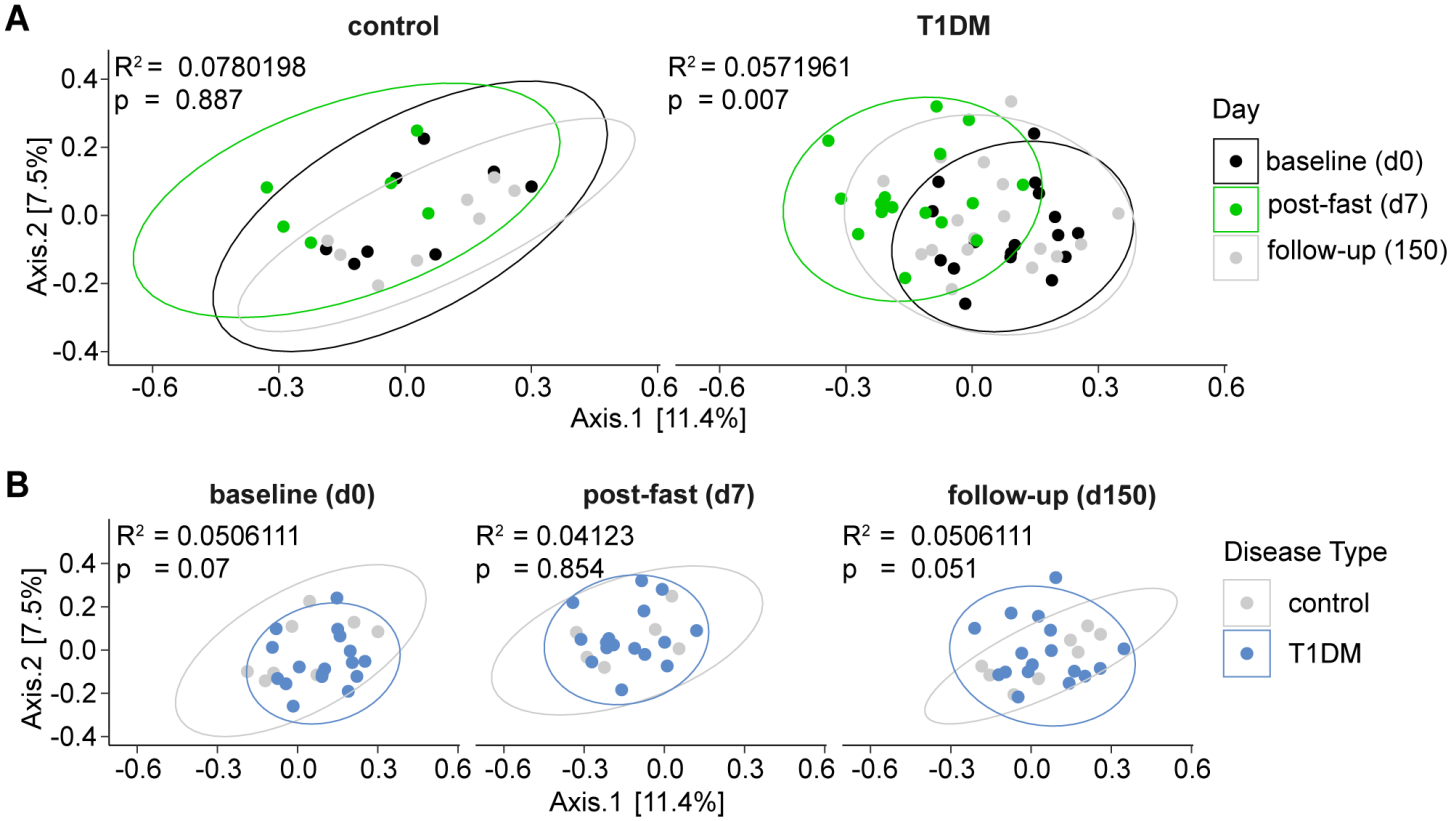
Post-fasting gut microbiome composition of T1D patients and controls converging. Principal Coordinates Analysis (PCoA) depicting bacterial β-diversity measured by Bray-Curtis dissimilarity. Statistical differences between groups were calculated by PERMANOVA. Ellipses represent 95% confidence intervals. **(A)** FAMED1 intragroup changes over time. Baseline (d0), post-fast (d7), follow-up (d150). **(B)** FAMED1 between-group differences at indicated time points. Statistical differences between groups calculated by PERMANOVA. Ellipses represent 95% confidence intervals.*** p < 0.0001, ** p < 0.001, * p < 0.01. p-values are adjusted for multiple comparisons using the Benjamini-Hochberg method.

To understand how the gut microbiome responds to fasting in T1D patients and controls, we analyzed all collected stool samples using 16S rRNA sequencing. Within the control group, no significant differences in overall microbiome composition (Bray-Curtis β-diversity distances) were detected between baseline, post-fast and follow-up samples. In contrast, within T1D patients, β-diversity differed significantly between timepoints (p = 0.007) (**Figure 2A**). When using a repeated measures design (considering n = 72 samples over all 3 time points) to compare the between-group (control vs. T1D) gut microbiome composition, we observed a trend towards differences in overall gut microbiome structure between the two groups at baseline (p = 0.07) and follow-up (p = 0.051) (**Figure 2B**), an observation already reported elsewhere (46, 47). Indeed, when calculating the effect only at baseline (n = 25 samples over all clinical variables), we did find that disease status (having T1D or not) significantly affected microbiome composition (p = 0.001), i.e. the gut microbiome composition of people with T1D differed from that of controls at baseline (**Supplementary Figure S1**), while no other tested clinical variable, including sex, age and BMI had a significant effect on baseline microbiome composition at this sample size (**Supplementary Figure S1**). Notably, immediately post fasting this difference in β-diversity between T1D patients and controls seemed to decrease as the gut microbiome composition of T1D patients and controls converge at this time point (p = 0.854) (**Figure 2B**). Taken together these findings indicate that fasting may have an equalizing effect on the gut microbiome of T1D patients and controls.

### Fasting induces short-term significant shift in microbial composition driven by changes in the relative abundance of several microbial taxa

**Figure 3:**
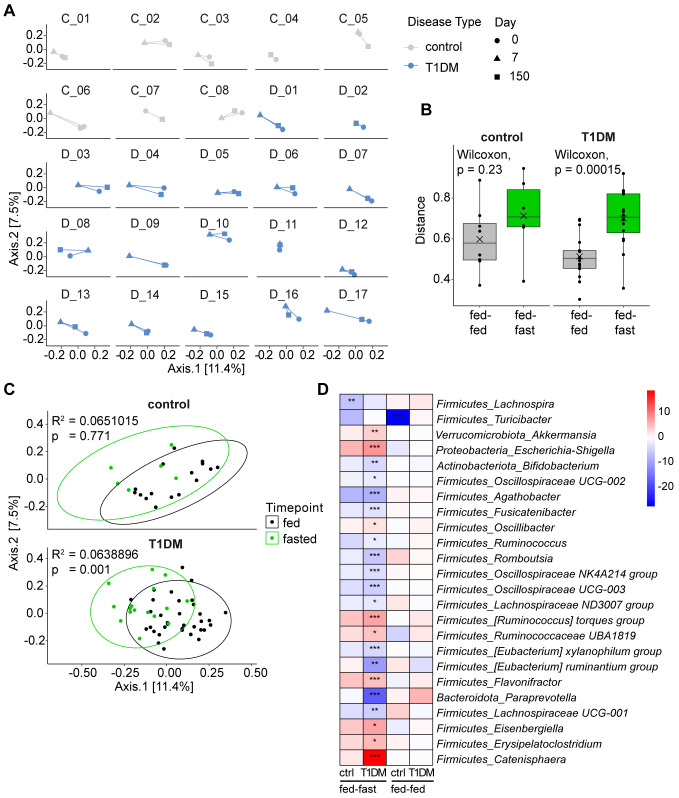
Fasting induces short-term significant shift in microbial composition. **(A)** PCoA plots showing Bray-Curtis dissimilarity by individual patients longitudinally. **(B)** Volatility analysis measuring distance between time points for individual patients using a matrix of Bray-Curtis distances. Fed-fed referring to distance between d0 and d150 (baseline and follow-up) and fed-fast referring to distance between d0 and d7 (baseline and post-fast). **(C)** PCoA plots showing Bray-Curtis dissimilarity grouped by nutritional state (“fed” considering samples from d0 and d150, “fasted” considering samples from d7). Statistical differences between groups calculated by PERMANOVA. Ellipses represent 95% confidence intervals. **(D)** Heatmap of differentially abundant taxa shown as the log_2_(fold-change) between time points for each significant genus. ***p < 0.0001, **p < 0.001, *p < 0.01. p-values are adjusted for multiple comparisons using the Benjamini-Hochberg method.

Looking at changes in microbial β-diversity longitudinally and for each fasting participant individually, it became apparent that the baseline and follow-up samples cluster closely together while the post-fasting samples are shifted away from both timepoints (**Figure 3A**). A volatility analysis confirmed that distances between samples from baseline to post-fast (fed-fast) were significantly longer than from baseline to follow-up (fed-fed) for T1D patients (p = 0.00015), indicating that a prolonged fast produces a dramatic, albeit transient change in gut microbiome composition (**Figure 3B**). This led us to group the baseline and follow-up samples together by nutritional status, considered “fed”, while the immediate post-fasting samples were considered “fasted”. Indeed, when only considering the nutritional status of the patients, we observed that fasting induced a significant shift in microbiome composition at the group level in T1D patients (p = 0.001) and a similar yet non-significant trend amongst controls (**Figure 3C**).

We next analyzed which microbiota members could be driving this compositional change and were significantly altered in their relative abundance in response to fasting. In the gut microbiota of T1D patients, we detected 21 differentially abundant genera following fasting. While none of the gut microbiota changes detected in controls reached significance, they did broadly follow the microbiota response pattern seen in fasted T1D patients in terms of direction and magnitude. For example, *Agathobacter*, *Fusicatenibacter* and *Oscillospiraceae UCG-003* were relatively decreased in both T1D patients and controls while *Escherichia/Shigella*, *Ruminococcus torques group* and *Ruminococcaceae UBA1819* were all increased in both T1D patients and controls (**Figure 3D**, **Supplementary Figure S2**).

When we included amplicon sequence variant (ASV) level data, a higher resolution approach approximating species level information, we detected differentially abundant ASVs that were specific to each group. For example, *Bacteroides vulgatus* and an ASV matching to *Prevotella* were only relatively increased in T1D patients but not in controls immediately post fasting, while *Roseburia intestinalis* along with several other ASVs was only relatively decreased in the control group but not in T1D patients (**Supplementary Figure S5B**). Comparing baseline to follow-up, we observed only one genus in the control group (*Turicibacter*) that was significantly decreased at 5–6 months post fasting. As such, fasting induced only transient changes to gut microbiome composition and was unable to shift the microbial composition to a new and stable long-term equilibrium.

### Fasting-induced microbiome changes correlate with changes in cardiometabolic risk markers over time

**Figure 4:**
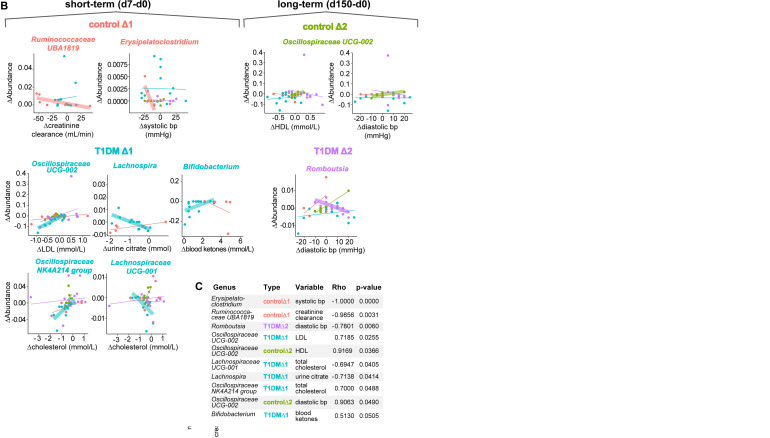
Microbiome differential abundance changes are correlated with changes in clinical parameters over time. **(A)** Heatmap colored by Spearman rank correlation coefficients (Rho) assessing association of significantly changed genera identified in **Figure 3D** with changes in clinical variables for the FAMED1 study. Cells colored in grey indicate that the standard deviation in one of the variables is zero, therefore a correlation coefficient cannot be determined. *p < 0.05. p-values are adjusted for multiple comparisons using the Holm method. **(B)** Correlation plots for the taxa significantly associated with clinical parameters identified in **Figure 4A**. Linear graphs highlighted using a fat stroke display the intervention group with the significant association. **(C)** Table displaying top ten most significant associations provided for reference.

In recent years, several causal relationships have been established for clinical parameters, such as blood glucose or ketone levels, driving specific microbiome changes (34, 48), as well as specific microbes precipitating changes in clinical variables e.g., *Akkermansia muciniphilia* improving insulin sensitivity, total plasma cholesterol and body weight (49). As part of the FAMED1 study, blood as well as urinalysis data were collected to monitor the safety of the dietary intervention (especially early detection and prevention of ketoacidosis), and to record improvements or deterioration of health markers including BMI, blood pressure, cholesterol, heart rate and HbA1c. We used the available data to correlate changes in these parameters immediately post-fasting and at follow-up, with the differentially abundant genera identified above (shown in **Figure 3D**, **Supplementary Figure S2**). After correcting for multiple comparisons, we found 9 significant associations between microbiota changes and clinical parameter changes (**Figure 4A**).

For example, the fasting-induced change in relative abundance of the genus *Oscillospiraceae - UCG-002* was found to be significantly associated with changes in LDL cholesterol levels in patients with T1D and with changes in HDL cholesterol levels and diastolic blood pressure in controls. Additionally, we found that the fasting-induced increases in the relative abundance of the genera *Erysipelatoclostridium* in controls and *Romboutsia* in T1D patients were correlated with a decrease in systolic and diastolic blood pressure, respectively. Our analysis also linked a relative increase in *Lachnospira* with a drop in urine citrate levels in T1D patients. In contrast, we found that changes in creatinine clearance, a measure for kidney function, were linked to modulations in the relative abundance of the genus *Ruminococcus UBA1819* in controls. Lastly, the tenth hit in our correlation analysis, though just missing the cut-off for significance at p = 0.0505, linked decreases in *Bifidobacterium* abundance with blood ketone levels in T1D patients. Thus, the majority (7 out of 9) of significant relationships detected between microbial abundance and clinical parameters involved alterations in either blood pressure or cholesterol levels correlating with changes in the abundance of microbes from the *Oscillospiraceae* or *Lachnospiraceae* families.

### Microbiome fasting signature shared between repeated fasts and different autoimmune diseases

Lastly, we investigated whether a prolonged fast modulates the gut microbiome of patients with different autoimmune diseases in a comparable manner or whether the resulting changes are disease-specific. To that end, we analyzed the gut microbiome of patients suffering from MS. We collected samples from a subgroup of the fasting arm of the Nutritional Approaches in Multiple Sclerosis (NAMS) study – a randomized, controlled, three-armed, single-center and parallel-group trial that took place at the same time as the FAMED1 study (fall of 2018) in the same geographic region (greater Berlin area) (50). In the NAMS study, MS patients were randomized into one of three dietary intervention groups: standard healthy diet, ketogenic diet, and a fasting intervention. In the latter group, MS patients underwent two seven-day fasting periods six months apart and adhered to an intermittent fasting schedule (14 h daily fast) in-between the longer fasts (51). Stool samples from the fasting arm were collected at baseline (day 0), at the 6 months follow-up (day 180) and immediately following both seven day fasting periods (day 7 and day 187) (**Supplementary Figure S3**). Baseline characteristics for the NAMS sub-cohort that provided stool samples are shown in **Table 1**. More details on participant recruitment, study design and diets for NAMS are provided in the methods section, in the published study protocol and clinical manuscript (50, 51).

**Table 1:** Baseline characteristics of MS patients in microbiome fasting study cohort.

Variables	Statistical Measures	Fasting group n = 10
Age (years)	**Mean** **(SD)**	44 (9)
Sex (female)	**n** **(%)**	9 (90)
MS duration since manifestation (years)	**Mean** **(SD)**	6.0 (4.9)
MS duration since diagnosis (years)	**Mean** **(SD)**	5.0 (5.0)
Disease modifying therapy (yes)	**n** **(%)**	6 (60)
BMI (kg/m^2^)	**Mean** **(SD)**	26.1 (4.9)
Blood glucose (mg/dl)	**Mean** **(SD)**	79.4 (8.8)
Insulin (mg/dl)	**Median** **(IQR)**	6.9 (8.4 - 4.6)
Leptin (µg/l)	**Median** **(IQR)**	7.0 (13.5 - 2.8)

**Figure 5:**
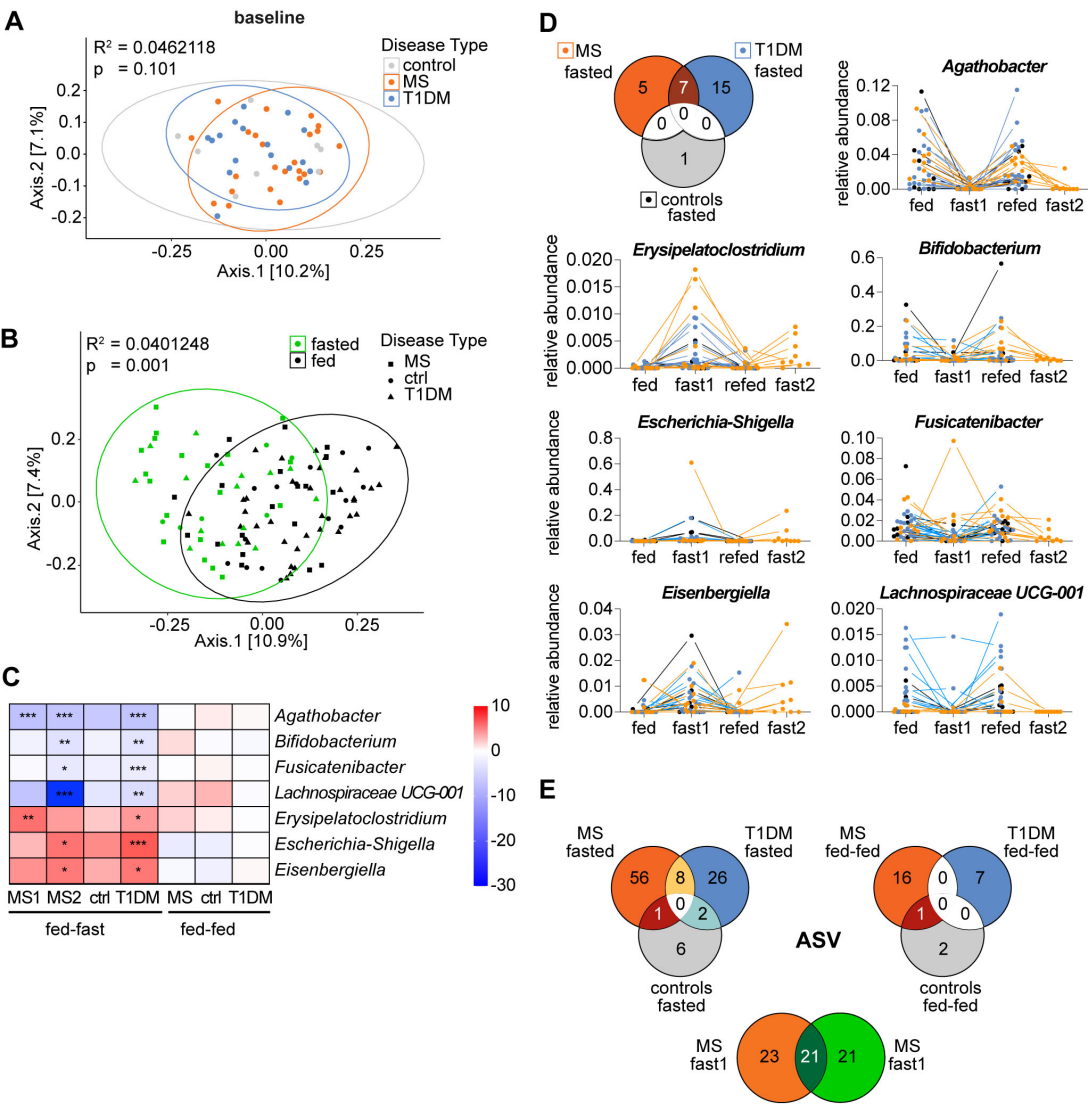
Differentially abundant genera form a microbiome fasting signature shared between the autoimmune diseases MS and T1D. **(A, B)** PCoA plots depicting bacterial β-diversity measured by Bray-Curtis dissimilarity considering all study participants from two studies **(A)** at baseline (d0) grouped by disease and **(B)** at all time points grouped by nutritional status with “fed” referring to combined samples from baseline and follow-up. Statistical differences between groups calculated by PERMANOVA. Ellipses represent 95% confidence intervals **(C)** Heatmap of the common differentially abundant taxa shown as the log_2_(fold-change) between time points for each genus that was significantly changed in both studies (same data as 3D and S4B but with standardized scale). ***p < 0.0001, **p < 0.001, *p < 0.01. p-values are adjusted for multiple comparisons using the Benjamini-Hochberg method. **(D)** Venn-Diagram of genera that are significantly changed immediately post-fasting compared to baseline (d0) in each group and change in relative abundance of the 7 conjointly changed taxa over time for all study participants. **(E)** Venn-Diagrams of common ASVs that are significantly changed in the respective group and time point.

In MS patients the first fast led to a significant decrease in relative abundance of the genus *Agathobacter* as well as a significant increase in 4 other genera including *Ruminococcus gnavus group* and *Hungatella*. Interestingly, the latter two as well as *Agathobacter* were changed in the same fashion along with seven additionally altered genera following the second fast in these patients (**Supplementary Figure S4**). In fact, we found substantial overlap with regards to the specific microbes changing in abundance following the two fasts in MS patients when we included amplicon sequence variant (ASV) level data. Specifically, approximately 50% of statistically differentially abundant ASVs were shared between the repeated fasts (significant ASVs baseline ➔ post-fast: n_(fast1)_ = 45; n_(fast2)_ = 42; n_(fast1∩fast2)_ = 21) as well as many more ASVs that were not significant in one of the two fasts but showed the same response pattern. For example, the ASVs matching *Eisenbergiella tayi*, *Erysipelatoclostridium* and *Subdoligranulum* were relatively increased following both fasts, but the increase was only significant following the first fast (**Figure 5E**, **Supplementary Figure S5A**).

Next, we compared the dissimilarity between gut microbiome composition of all groups (T1D, MS, control) at baseline (d0). Larger studies and meta-analyses have reported that patients suffering from autoimmune diseases harbor a distinct gut microbiome that differs from that of controls in β-diversity and can be characterized by disease-specific changes of several bacterial taxa (8, 52). However, we found no clusters separating MS patients, T1D patients or their controls at baseline with regards to β-diversity (**Figure 5A**).

When the overall changes in β-diversity of all samples were assessed based on nutritional status, we did however detect a collective and significant microbiome shift induced by fasting (**Figure 5B**), indicating commonalities in the microbiota’s response to fasting for all groups.

Indeed, we identified 7 genera that were conjointly and significantly changed in the MS and T1D patients post-fasting (**Figure 5E**). That corresponds to 33% (7 out of 21 genera) of all differentially abundant changes in T1D patients and 40% (2 out of 5 genera) for the first and 70% (7 out of 10 genera) for the second fast in MS patients.

We found that fasting led to a relative decrease of the taxa *Agathobacter, Bifidobacterium, Fusicatenibacter and Lachnospiraceae UCG-001* as well as an increase in relative abundance of *Erysipelatoclostridium, Escherichia/Shigella* and *Eisenbergiella*. Intriguingly, the directional changes for these 7 bacterial taxa following repeated fasts, was identical in both patient groups, and in the control group. Moreover, the magnitude of changes for these taxa was comparable between groups (**Figures 5C, D**). A similar picture emerged from ASV level analysis, showing that MS and T1D patients shared 8 differentially abundant ASVs post-fasting that all responded to the intervention in the same way: A relative decrease was noted in *Bifidobacterium adolescentis/faecale/stercoris* and *Romboutsia ilealis/timonensis* as well as in two ASVs matching to *Agathobacter* and in one ASV matching to a non-annotated Firmicutes member. Further, *Escherichia-Shigella*, *Merdibacter* and *Lachnoclostridium* were all relatively increased (**Supplementary Figure S5** – highlighted text). In addition, multiple closely related, but non-identical ASVs that were subsequently assigned to the same genus/species (indicating strain level differences) were modulated by fasting in a comparable manner across groups – e.g. the ASVs assigned to *Ruminococcus torques group* were relatively increased following fasting in all participants while the ASVs assigned to *Faecalibacterium prausnitzii* were decreased. Altogether about ~25% of ASVs were regulated by fasting in a similar fashion. Taken together, this suggests that a portion of the fasting-induced microbiome changes are independent of host disease status and represent a microbiome fasting signature.

## Discussion

In this study, we show that following fasting, the gut microbiome composition of T1D patients and controls converge. Furthermore, we find that fasting induces a collective change in the composition of the gut microbiome of T1D patients, MS patients, and controls. Indeed, fasting induced multiple overlapping, differentially abundant bacterial genera in both patient groups suggesting the existence of a partially disease-independent microbiome fasting signature. Finally, we found that the fasting-mediated differential abundances of several taxa from the *Oscillospiraceae* and *Lachnospiraceae* families correlated with fasting-induced changes in blood pressure and cholesterol markers in T1D patients and controls.

The shared fasting-induced microbiome changes we detected concur with a growing body of existing research on the profound effects of fasting on the gut microbiome. For example, the fasting-induced depletion of the genera *Agathobacter*, *Fusicatenibacter* and *Lachnospiraceae UCG-001*, all members of the *Lachnospiraceae* family, have been previously described (40, 53). Specifically, the drop in *Agathobacter* relative abundance was the most consistent change across all groups post fasting in our study. Similarly, previous studies have identified *Eubacterium rectale*, recently reclassified into *Agathobacter rectalis*, as a species that is significantly and robustly diminished in abundance in response to prolonged fasting (41, 54, 55). This likely reflects the fact that *Agathobacter* spp. are some of the most abundant butyrate producers in the human gut and often associated with a fiber-rich diet (54). In fact, many members of the *Lachnospiracea* family are known to be specialized in the degradation of complex plant polysaccharides and therefore susceptible to fiber-deprivation during fasting (54, 56).

It should be noted that the metabolic capabilities in this family are heterogeneous as other *Lachnospiracea* genera in our study were increased post fasting - including *Eisenbergiella*, *Hungatella*, *Ruminococcus gnavus group* and *Ruminococcus torques group* – all of which were also increased in abundance in response to fasting in healthy fasting participants (40, 53). Notably, the latter two *Ruminococcus* groups are well known host mucin-degraders and *Hungatella hathewayi* is an efficient glycosaminoglycan degrader. As such, their collective increase illustrates a switch towards consumption of host-derived energy substrates (57–59). Interestingly, the species *Eisenbergiella massiliensis* is increased in mice placed on a ketogenic diet and highly correlates with serum β-hydroxybutyrate in humans (60). Furthermore, isotope tracing studies revealed that gut microbes can feed on circulating host β-hydroxybutyrate as it is one of the few host nutrients able to reach the intestine via monocarboxylate transporters (61). Given the physiologic increase in β-hydroxybutyrate during fasting, it appears reasonable to assume that some of the microbial members that increase in abundance in response to fasting, including *Eisenbergiella*, may feed on this ketone body.

Aside from the taxa that were conjointly changed in MS and T1D patients, we also found overlap with the literature for fasting-induced changes in differentially abundant taxa that, in our study, were specific for each disease (MS and T1D, ASV level): For example, increases in the relative abundance of *Phascolarctobacterium, Blautia, Butyricimonas, Bacteroides fragilis, Flavonifractor plautii*, and decreases in *Ruminococcus bicirculans, Roseburia intestinalis, Faecalibacterium prausnitzii, Romboutsia ilealis/timonensis, Coprococcus eutactus, Fusicatenibacter saccharivorans, Lachnospira pectinoschiza* and *Dialister* have all been previously reported in other fasting studies with either healthy participants or with other patient populations (metabolic syndrome, heart disease) (39–41, 62). Furthermore, we found that the fasting-induced microbiome changes in controls follow the same pattern as the T1D and MS patients, and although none of the changes were significant in the control group in our study, (likely due to the small group size), larger studies in healthy participants showed significant changes for the respective microbes (40, 44).

Taken together, it is remarkable how consistent our findings are with previously published research, given that microbiome composition is highly individualized and given the differences in study population, fasting protocols, modes of sample collection, DNA extraction, sequencing and analysis. This suggests two things: Firstly, that a more comprehensive dataset with bigger sample size would likely uncover more fasting-induced conjointly regulated taxa across healthy individuals and patients and that therefore, the gut microbiome response to fasting may be not partially but rather largely disease-independent.

Secondly, this indicates that the gut microbial response to fasting may be relatively conserved. Indeed, studies examining the gut microbiome of different animals after prolonged fasts and hibernation periods also indicate several shared/generalizable microbiome responses to fasting (44, 63). The conserved fasting response might reflect a forced functional diversification of the gut microbiota under the pressure of nutrient deprivation, whereby one side of the coin is how the host keeps its microbiota alive: We and others observed that during fasting taxa which mainly degrade dietary substrates decrease in abundance, while taxa that are known to be metabolically flexible and able to utilize host-derived nutrients, such as mucolytic bacteria (i.e., *Ruminococcus gnavus/torques, B. fragilis/vulgatus, Akkermansia muciniphila*) increase in abundance (40, 43, 44, 64). This can be resolved down to the level of genomic potential, as has been demonstrated for bacterial-encoded carbohydrate-active enzymes (CAZymes): Fasting specifically depletes dietary fiber-digesting CAZymes and enriches mucin and glycosaminoglycan-metabolizing CAZymes to the extent that fasting-induced changes in a given gut microbiome can be predicted with high accuracy purely based on the genomic CAZyme potential (53).

The other, as of yet underexplored side of the coin, may be how the microbiota help to keep their host alive: Metagenomic pathway analysis shows that the ketone synthesis and degradation metabolism pathway is increased within the gut microbiome of fasted mice (37). In line with this is another study showing that liver ketogenesis is reduced in fasted germfree mice (65). This suggests that, next to utilizing β-hydroxybutyrate for their own nutrition, the gut microbiota could play an important role in augmenting host ketone production during periods of fasting.

Since most changes of the fasted gut microbiome do not seem to be specific to T1D or MS patients it remains speculative how rather conserved fasting-induced gut microbiome changes could mechanistically be linked to improvements in the inflammatory components of type 1 diabetes and other auto-immune diseases. One possible avenue could be via the consistent depletion of *Bifidobacterium* across all fasting groups. Ketogenic diets, specifically β-hydroxybutyrate, have been shown to deplete *Bifidobacteria*, an effect reported to be responsible for a decrease in intestinal Th17 cells (34). In fact, the tenth hit in our clinical correlation analysis of the FAMED1 cohort linked shifts in blood ketone levels with changes in *Bifidobacterium* abundance in T1D patients. The *Bifidobacteria* depletion we and others observe post-fasting may thus also be mediated by the fasting-induced increase in β-hydroxybutyrate and contribute to improved Th17 homeostasis, and therefore reduced inflammation, in T1D and possibly MS patients (39, 41, 62).

Another mechanism could be related to the relative increased abundance we observed for *Escherichia/Shigella* (66) and *Erysipelatoclostridium*, likely *Erysipelatoclostridium ramosum*, (also known as *Clostridium ramosum*, to be renamed *Thomasclavelia ramosa* (67)), which have both previously been reported as increased in the context of fasting (40, 41). Both taxa are implicated in promoting proinflammatory responses and contain species that have been classified as opportunistic pathogens (68, 69). Furthermore, both taxa have been associated with MS pathology and *Escherichia-Shigella* is found enriched in the gut microbiome of children with new-onset T1D (70, 71). As such, the presence of these taxa may predispose individuals to heightened immune responses, fostering an environment liable to trigger or exacerbate MS-related neuroinflammation as well as increased β-cell destruction. The observed further increase of these bacteria during fasting could, instead of exacerbating inflammation, potentially lead to the creation of a more tolerogenic response profile as cues from the host environment always contribute to scale and intensity of the immune reaction e.g., effector T cell function/differentiation is diminished under nutrient deprivation while Treg cells are less impacted by it (72, 73).

While we did not detect any links to primary disease-related outcomes in the fasting-induced gut microbiome changes in patients with T1D (such as insulin dose or glucose levels), we did detect that changes in certain microbiota members correlated with cardiometabolic risk markers (blood pressure and cholesterol levels). These parameters play a key role in diabetic co-morbidities such as metabolic syndrome which is increasingly recognized as a negative factor contributing to disease severity and progression in T1D (74). Indeed, multiple studies have shown that the most frequently reported clinical outcomes of fasting are improvements in blood pressure and optimization of cholesterol profiles (26, 41, 75, 76). We identified several genera associated with cholesterol or blood pressure changes that belong to the *Oscillospiraceae* family. In previous studies, the genus *Oscillospiraceae UCG-002* was negatively associated with insulin resistance (HOMA-IR index) while *Oscillospiraceae* as a family were shown to be negatively correlated with LDL specifically. Notably, one genus of the *Oscillospiraceae* family, namely *Oscillibacter*, which was significantly increased in abundance post fasting in the T1D group (and at ASV level in the first fast of the MS group), has been associated with decreased fecal and plasma cholesterol levels. In fact, *Oscillibacter* spp. were found to possess conserved cholesterol-metabolizing enzymes (77), thus lending themselves to the possibility to contribute to the fasting-induced decrease in cholesterol levels by consuming cholesterol as substrate and plausibly linking this genus and/or the respective family to metabolic and cardiovascular health status (78, 79). Furthermore, changes in relative abundance of the genera *Erysipelatoclostridium* and *Romboutsia*, which we linked to systolic and diastolic blood pressure changes respectively, have been found associated with these parameters before (80, 81).

Kidney function, which is often impaired in patients with T1D, has been reported to also be affected by the gut microbiome, either directly, through their uremic metabolites, or via microbiome-mediated changes in patient blood pressure, immune response and metabolism (82, 83). Our analysis showed a negative correlation between fasting-induced changes in *Lachnospira* and citrate levels in the urine of T1D patients. Patients with decreased citrate urine levels are at an increased risk for kidney stone formation. An increased representation of *Lachnospira* has been previously linked with a heightened risk for lithiasis (84, 85). We also found that changes in creatinine clearance were associated with fasting-induced modulations of *Ruminococcus UBA1819* in controls. Notably, in previous studies, children with primary nephrotic syndrome who relapsed were shown to have a higher proportion of *Ruminococcus UBA1819* than non-relapsing children (86). Whether these fasting-induced changes in the gut microbiome are causally linked to the observed clinical effects remains to be tested.

Most of the microbiome alterations (β-diversity and differential abundance) observed directly after fasting in T1D and MS patients, had reverted to baseline by the 5 and 6 months follow up. Likewise, previous studies report a return to baseline microbiome composition as early as 1 and 3 months after a fasting intervention of healthy individuals over 10 d, resp. of patients with metabolic syndrome over 5 d (40, 41, 53). This “dietary intervention-resilient gut microbiome” has been described in many studies (87, 88). Given the diverse and important functions performed by the gut microbiome, its typical resilience against perturbations (i.e., through infections, food toxins, medication) is likely beneficial from an evolutionary perspective. However, when the microbial community in an individual has been configured to elicit or worsen disease, as described for both MS and T1D patients (8), this resilience may become problematic and potentially promote disease chronicity. Whether the transient but dramatic fasting-induced gut microbiome change translates into long-term clinical benefits should be explored in future studies, along with surveying the gut microbiome during the re-feeding period in short intervals to determine how quickly the microbial community rebounds.

The main limitations of this study are its relatively small sample size and mode of sequencing. Microbiome changes were declared as secondary endpoints for both studies and were therefore not powered specifically for this study. Rather, this study serves as an exploratory analysis. Secondly, the reliable resolution of 16S rRNA sequencing is capped at the genus level. As it is a non-quantitative method, relative abundance estimation does not always correlate perfectly with total microbial abundance. Thirdly, the generalizability of the study may be limited as the study population was largely European, being recruited from two academic centers in Germany. In contrast, the strengths of our study include our longitudinal data, for example in the NAMS fasting group where individual patients served as their own control and underwent repeated fasts. Furthermore, both studies (NAMS and FAMED1) were undertaken under similar conditions in the same geographic location with very similar fasting protocols, and a unified downstream analysis was employed (DNA collection, extraction, sequencing, analysis), thereby reducing potential variability that could have otherwise confounded the results.

In summary, we provide evidence that fasting has a potentially conserved signature effect on gut microbiota composition that is largely independent of disease status. These microbiota changes are likely driven by the need of the gut microbial community to diversify their nutrient sources when dietary food is absent. Moreover, our data supports previous studies linking fasting-induced changes in several bacterial taxa with fasting modulation of blood pressure and cholesterol markers. Hence, future studies should aim to tease apart whether reported associations between bacteria and clinical parameters are responsible for mediating any of the fasting-induced benefits, using gnotobiotic mouse models for example. Different lengths of fasts (intermittent, days, weeks) and protocols (water, juice, and other formulations including fiber) should be compared directly to determine if the effects of fasting on the gut microbiome are dependent on any of these factors. Lastly, we do not address the potential effect that fasting may have on the gut virome and mycobiome. Both viruses and fungi have been implicated in autoimmune disease pathology (89, 90), and increased risk and severity of yeast and viral infections are a major co-morbidity burden in T1D patients (91). Thus, fungal and viral communities should also be profiled in future fasting studies.

## Methods

### Study cohorts

The feasibility pilot study of fasting for patients with T1D (FaMeD1, Fasten für Menschen mit Typ 1 Diabetes) was registered in the German Trial register under the accession number DRKS00017504 and ethical approval was obtained by the Ethics Committee of Witten/Herdecke University. The Nutritional Approaches to Multiple Sclerosis (NAMS) study was registered on ClinicalTrials.gov under the accession number NCT03508414. Ethical approval was given by the institutional review board of Charité - Universitätsmedizin Berlin. Written informed consent was given by the participants and trials were conducted in accordance with the principles of the Helsinki Declaration.

For the FAMED1 study, twenty T1D patients and ten healthy controls, recruited from three study sites in Germany, were enrolled into a seven-day multimodal fasting intervention in an in-patient, non-hospital setting near Berlin, Germany. On average 200 kcal/d (max 250 kcal/d) were given predominantly as carbohydrates by different high-quality fruit and vegetable juices, filtered vegetable broth or oat gruel. Information supplied by the manufacturer for juices (Voelkel GmbH, Höhbeck, Germany) state minimal amounts of proteins (max. 0.7 g/100g), fats (max. 0.2 g/100g) or fibers (max. 1.0 g/100g). Similarly, oat gruel mainly consists of easily digestible carbohydrates (11.25g/100 ml) with minimal fiber, protein and fat (92). Vegetable broth as well as any tea used did not contain any energy nor fibers. As such, on average the macronutrient composition of the fasting diet consisted of approximately 85-90% carbohydrates, 0-5% fat, and 5-10% protein, with minimal fiber content. Calories were slowly decreased before the fast, while calories and solids were slowly increased after cessation of the fast. Patients were followed up 4–5 months later. Participants collected their fecal samples before and after the fast as well as at the follow-up time point. Blood ketones and glucose were monitored daily during the fast using a handheld device (GlucoMen Areo 2K). For urinary analyses, urine was collected over 24h three times – pre-, during and post-fasting. Blood samples were taken at baseline, post-fast and at follow-up for measurement of clinical parameters including total cholesterol, high-density lipoprotein (HDL), low-density lipoprotein (LDL) and submitted to the hospital laboratory of Immanuel hospital, Berlin. Glycated hemoglobin (HBA1c) was individually reported for patients before and after intervention from their primary physician’s office. More details on the study design, inclusion/exclusion criteria as well as the main results of the study can be found in previously published sources (26, 45, 93).

For the NAMS study, stool samples were collected from a sub-cohort of 26 MS patients recruited from all parts of Germany, who were randomized into one of three nutritional intervention groups (in this manuscript only data from group 3 – the fasting intervention was used) (1): Patients followed a diet recommended by the German nutrition society (DGE) modified to a 5:1 ratio of omega-6 to omega-3 fatty acids (control group) (2); Participants consuming a ketogenic diet containing approximately 70-80% fat, 15-20% protein and 5-10% carbohydrates, targeting and recording serum β-hydroxybutyrate levels of 0.5-3.0 mmol/L (3); Patients followed a fasting diet comprised of two periods of seven-day fasts six months apart. In-between prolonged fasts, the participants were advised to adhere to the same diet as the control group, albeit with an added intermittent fasting schedule (14h daily fast). During the seven-day fasting periods, MS patients were consuming a diet very similar to participants in the FAMED1 study, ie. low-caloric vegetable broth and juice amounting to no more than 400 kcal/d with unrestricted amounts of herbal teas and water (**Supplementary Table S1**).

Patients were counseled for all three interventions in a group setting but interventions were undertaken individually in a non-centralized, at-home environment. At the initial visit, clinical parameters were recorded and blood samples submitted to *Labor Berlin (*central laboratory of Charité – Universitätsmedizin Berlin) for analysis of serum insulin, leptin and blood glucose. Dietary compliance was monitored by ensuring attendance of dietary sessions (every second day during fasting periods and additionally in between study visits).

### Microbiome analysis

#### Stool collection and DNA extraction

All study participants were given OMNIgene^®^•GUT collection tubes (OM-200, Genotek) with detailed instructions on how to self-collect stool. After stool collection, tubes were handed to the study nurses and subsequently frozen and stored at -80°C until all study time points from all participants had been collected. All samples were then shipped together at RT to the Children’s Hospital of Philadelphia (CHOP) Microbiome Center for DNA extraction and sequencing. DNA was extracted using the DNeasy PowerSoil Kit (Qiagen) following the manufacturer’s instructions, incorporating the optional heating step at 70°C for 10 min before bead beating.

#### Library prep and 16S rRNA sequencing

A two-step PCR was chosen for library generation. For the first reaction, the 16S V3V4 region was amplified in 25 µl quadruplicates. The Q5 Hot Start High-Fidelity DNA polymerase (New England BioLabs) was used with a primer pair first tested and characterized by Klindworth et al. (94) including overhanging Illumina adapter sequences (more detailed information and protocol steps can be found here:

https://support.illumina.com/documents/documentation/chemistry_documentation/16s/16s-metagenomic-library-prep-guide-15044223-b.pdf)

The quadruplicate reactions were then pooled and the DNA was cleaned using a 1:1 ratio of solid-phase reversible immobilization (SPRI) on carboxylated paramagnetic beads (GE Healthcare, 65152105050250). For the second PCR reaction, Nextera XT barcodes (Illumina) were added. Libraries were cleaned again, using SPRI beads. The amount of nucleic acid was quantified with PicoGreen™ (P7589, Invitrogen, ThermoFisher Scientific). Equimolar amounts of respective samples were pooled and sequenced on the Illumina MiSeq using 2x300 bp chemistry (MiSeq Reagent Kit v3 MS-102-3003, Illumina).

### Microbiome computational and statistical analysis

Illumina sequencing data were processed and analyzed using a custom R pipeline (version 4.2.2) based on the R package dada2 (version 1.20.0) with the help of Gut4Health (RRID: SCR_023673) (95). Reads were demultiplexed using bcl2fastq (version 2.20.0.422). Primer and adapter sequences were removed from raw fastq files using cutadapt (version 1.18) (96). Low-quality reads were filtered with the filterAndTrim function by removing forward and reverse reads with expected error (EE = sum(10^(-Q/10)) greater than 3 and 5, respectively. Resultant reads were denoised and merged using dada2 and a table of ASV (amplicon sequence variants) was generated. Taxonomic classifications were assigned to ASVs using the SILVA database (version 138) (97). Samples with fewer than 1,000 ASV-assigned sequences as well as ASVs present in less than 5% of samples were omitted. Phyloseq v1.38.0 (98) was used for further statistical analyses: β-diversity calculations were performed using Bray-Curtis distance matrices and visualized as PCoA plots or volatility plots. Differences in overall composition between groups as well as associations of baseline clinical variables with gut microbiome composition were assessed using PERMANOVA. Differential abundance was calculated using DESeq2 (99) on variance-stabilizing transformed counts utilizing a Wald test to determine significance and adjusting for multiple comparisons with the Benjamini-Hochberg method. Associations between changes in differentially abundant taxa and changes in clinical variables over time were assessed by Spearman rank correlation with the Holm method adjusting for multiple comparisons.

## Data Availability

The datasets presented in this study can be found in online repositories. The names of the repository/repositories and accession number(s) can be found below: https://www.ncbi.nlm.nih.gov/, PRJNA967360.
