# Supplementary material for: Fasting elicits gut microbiome signature changes that extend to type 1 diabetes patients
Source: Front Endocrinol (Lausanne). 2025 Aug 13;16:1623800. doi: 10.3389/fendo.2025.1623800 (PMC12380539; doi:10.3389/fendo.2025.1623800)
Supplement: Supplementary file 1 [file DataSheet1.docx]

**Supplementary Tables and Figures**

**Table S1:**

**Dietary intake data for NAMS standard diet, fasting group and ketogenic diet**

| **FASTING GROUP** | **during eating periods** | | **during fasting** |
| --- | --- | --- | --- |
| **all data as mean (SD)** | **Baseline d0**  **(n = 28)** | **9 months**  **(n = 26)** | **Day 1-7 or 181-187** |
| **Energy (kcal)** | 1738 (419) | 1560 (537) | ~ 400 |
| **Fat (%)** | 40 (11) | 38 (8) | ~ 0 - 5 |
| **Fat (g)** | 81 (35) | 67 (27) | ~ 0 - 2.2 |
| **Carbohydrate (%)** | 39 (11) | 41 (8) | ~ 85 - 90 |
| **Carbohydrate (g)** | 144 (71) | 150 (70) | ~ 85 - 90 |
| **Protein (%)** | 15 (3) | 15 (3) | ~ 5 - 10 |
| **Protein (g)** | 65 (20) | 56 (20) | ~ 5 - 10 |
| **Fiber (g)** | 22 (8) | 21 (11) | ~ 0 - 4 |

| **STANDARD DIET GROUP** |  | |
| --- | --- | --- |
| **all data as mean (SD)** | **Baseline**  **(n = 25)** | **9 months**  **(n = 20)** |
| **Energy (kcal)** | 1923 (400) | 2042 (1301) |
| **Fat (%)** | 36 (6) | 35 (10) |
| **Fat (g)** | 78 (22) | 72 (18) |
| **Carbohydrate (%)** | 43 (7) | 44 (12) |
| **Carbohydrate (g)** | 172 (89) | 211 (298) |
| **Protein (%)** | 15 (3) | 15 (3) |
| **Protein (g)** | 73 (19) | 72 (24) |
| **Fiber (g)** | 23 (8) | 33 (19) |

| **KETOGENIC DIET GROUP** |  |  |
| --- | --- | --- |
| **all data as mean (SD)** | **Baseline**  **(n = 24)** | **9 months**  **(n = 23)** |
| **Energy (kcal)** | 1811 (455) | 1895 (548) |
| **Fat (%)** | 43 (9) | 63 (10) |
| **Fat (g)** | 89 (39) | 136 (52) |
| **Carbohydrate (%)** | 36 (9) | 15 (10) |
| **Carbohydrate (g)** | 136 (71) | 57 (50) |
| **Protein (%)** | 16 (4) | 18 (4) |
| **Protein (g)** | 72 (22) | 83 (25) |
| **Fiber (g)** | 25 (13) | 24 (8) |

**
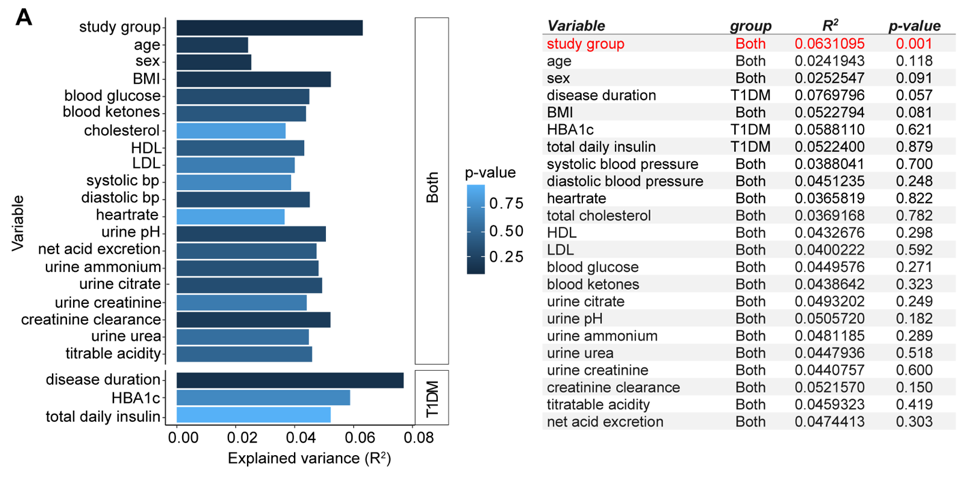
Figure S1**

**Figure S1. No significant effect of clinical variables on microbiome composition at baseline.**

**(A)** Percentage of variation in overall gut microbiome composition explained by each clinical variable at baseline (d0) for FAMED1 study. Variables for which that proportion (R2) is significant are highlighted in red in tables where exact values are provided for reference. ** p < 0.001. Statistical significance determined by PERMANOVA. Tables with exact values is provided for reference. Related to Figure 2.

**
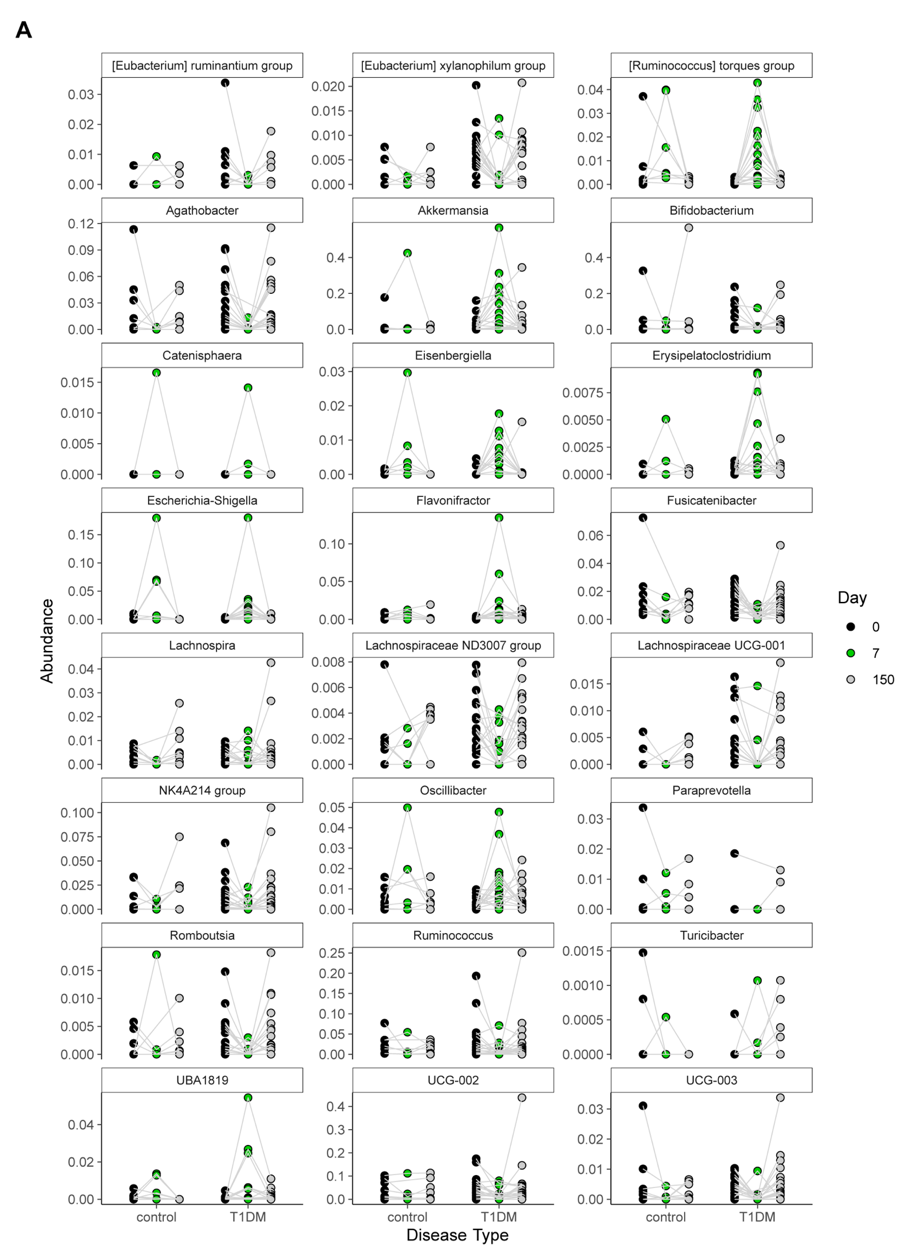
Figure S2**

**Figure S2. All differentially abundant genera induced by fasting in the FAMED1 cohort.**

**(A)** Change in relative abundance over time for all genera significantly changed by the intervention and shown in Figure 3D for FAMED1 study. Related to Figure 3.

**
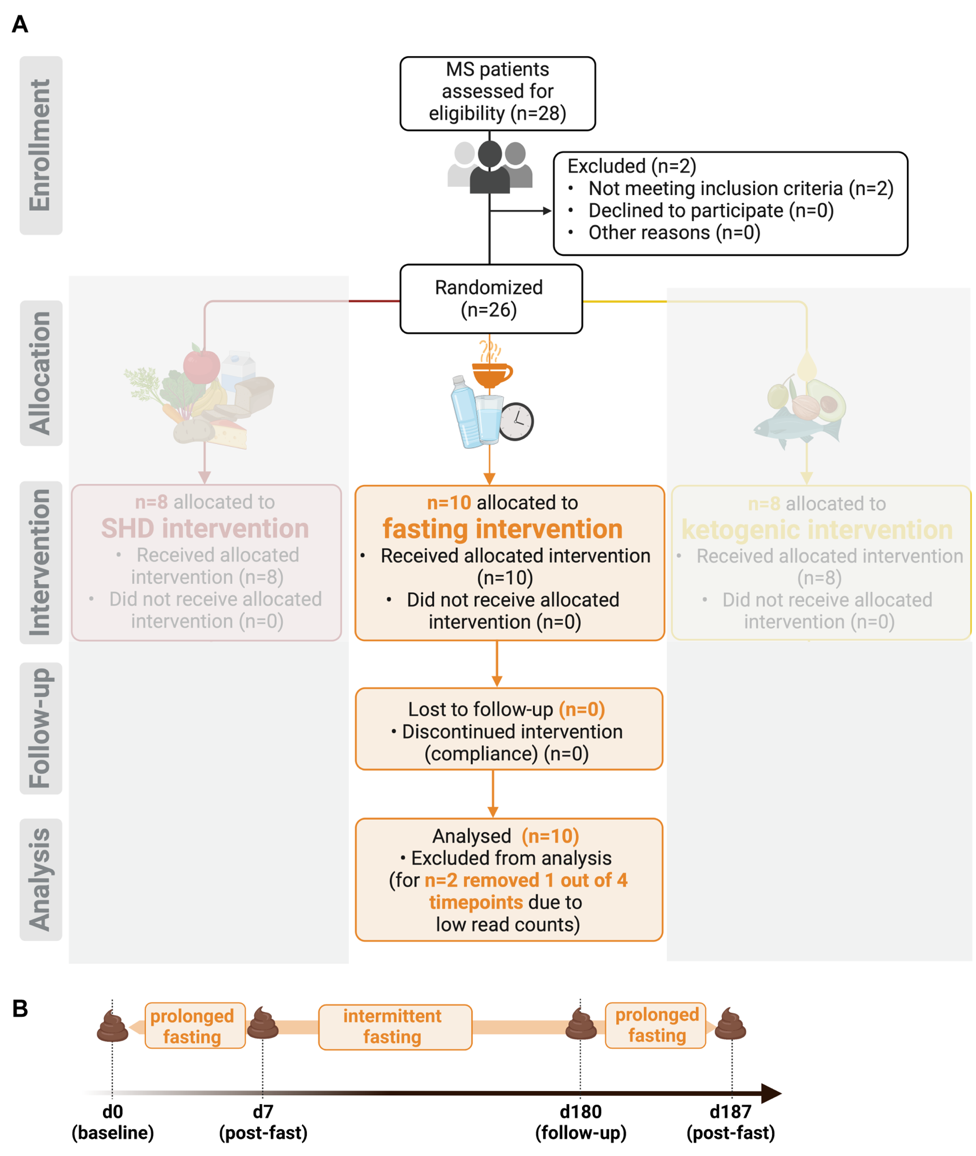
Figure S3**

**Figure S3. Overview of study design NAMS study.**

**(A)** Design of the Nutritional Approaches in Multiple Sclerosis (NAMS) study as a randomized, controlled, three-armed, single-center and parallel-group study. Only data from the fasting arm was considered for this manuscript. **(B)** Timeline of stool sample collection. Figure created with BioRender.com.

**
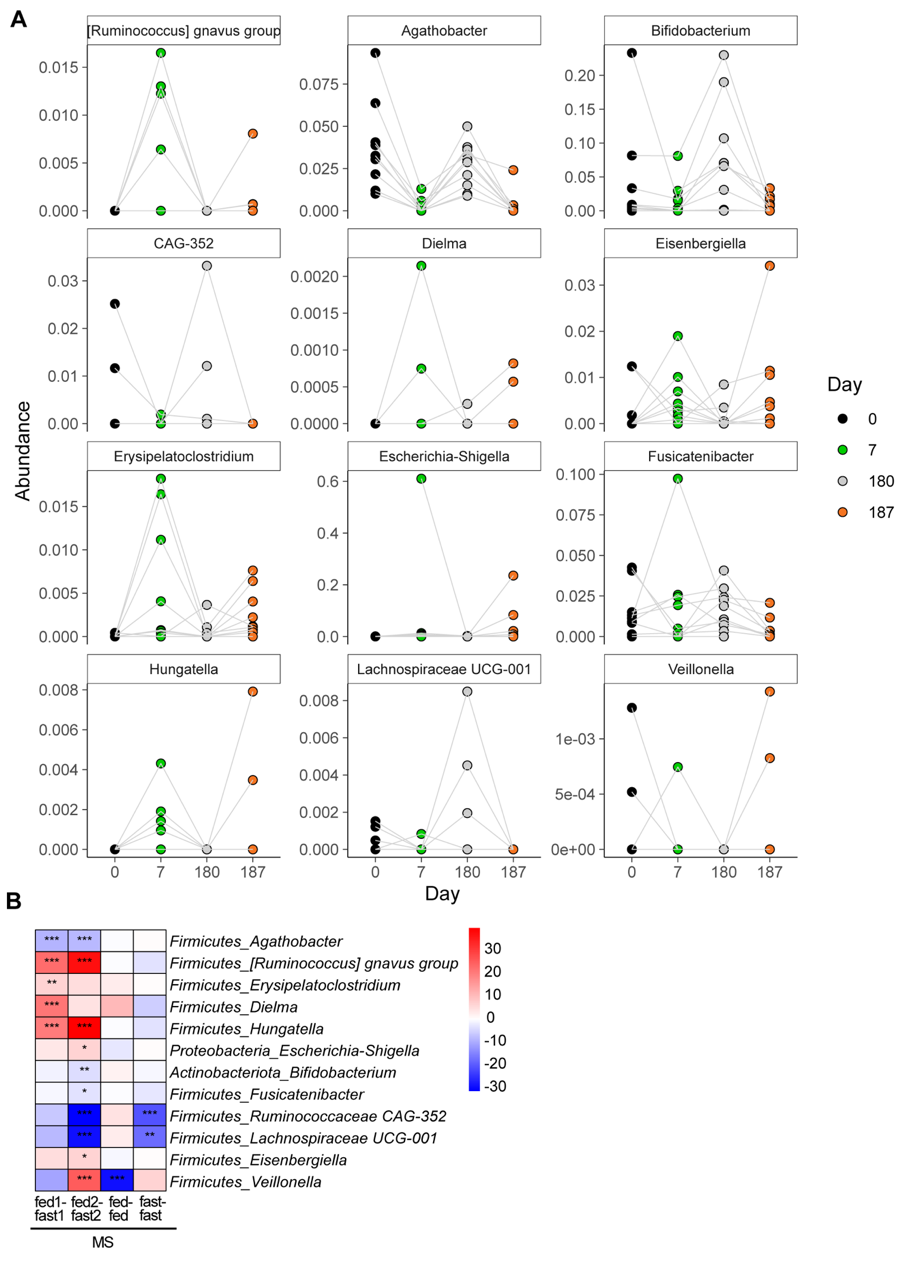
Figure S4**

**Figure S4. All differentially abundant genera induced by fasting in the NAMS cohort**

**(A)** Change in relative abundance over time for all genera significantly changed by the intervention for NAMS study. **(B)** Heatmap of differentially abundant taxa shown as the log_2_(fold-change) between time points for each significant genus in the NAMS study. *** p < 0.0001, ** p < 0.001, * p < 0.01. p-values are adjusted for multiple comparisons using the Benjamini-Hochberg method. Related to Figure 5.

**
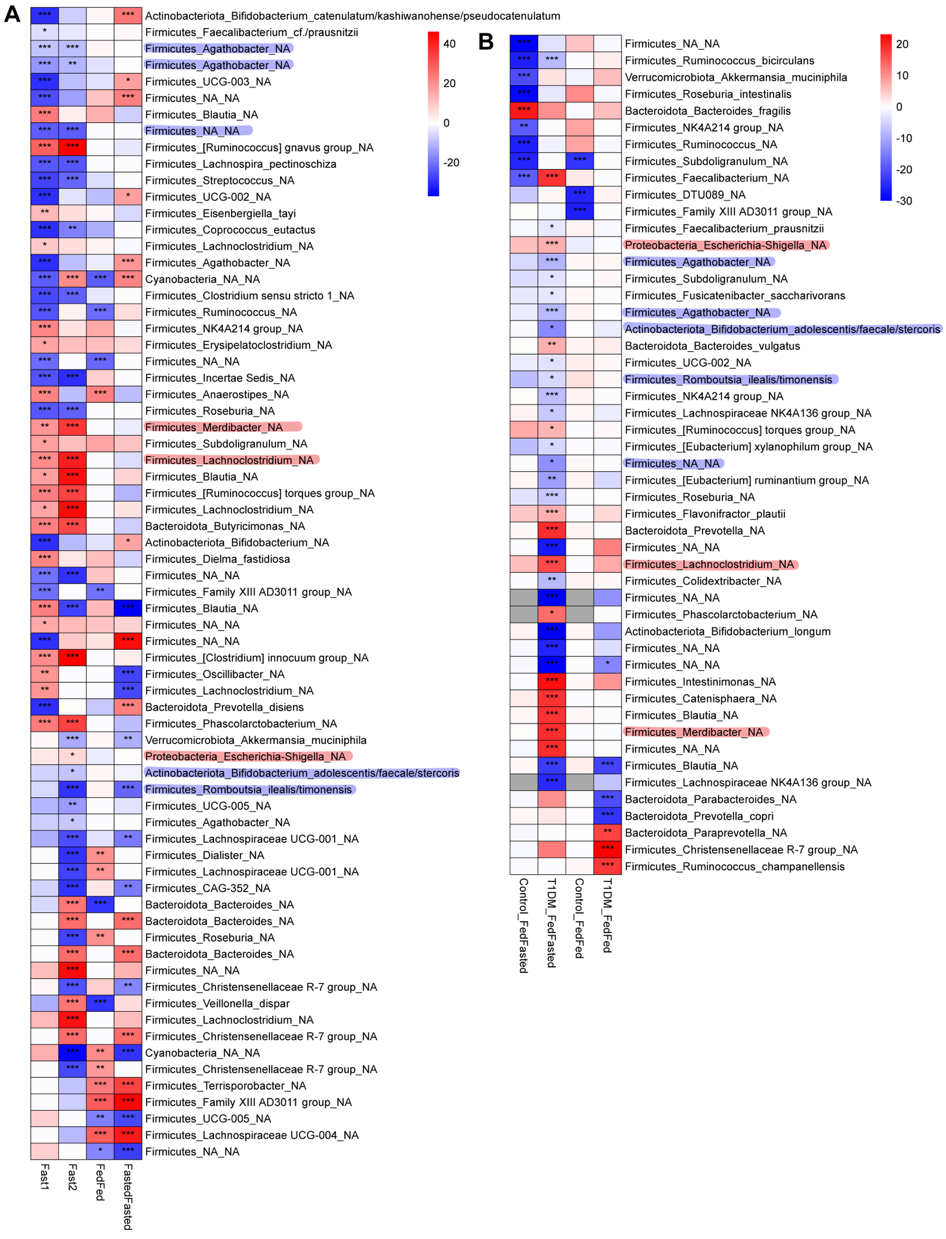
Figure S5**

**Figure S5. Differentially abundant amplicon sequence variants (ASVs) induced by fasting**

**(A, B)** Heatmap of the significant differentially abundant ASVs shown as the log_2_(fold-change) between time points for **(A)** the NAMS fasting group (Fast1: d0-d7; Fast2: d180-d187; FedFed d0-d180; FastedFasted: d7-d187) and **(B)** for both groups of the FAMED1 study. Conjointly changed ASV following fasting highlighted in blue (decreased) and red (increased). *** p < 0.0001, ** p < 0.001, * p < 0.01. p-values are adjusted for multiple comparisons using the Benjamini-Hochberg method. Related to Figure 5.
